# Automated Stereotactic Gamma Ray Radiosurgery to the Pituitary Gland in Terminally Ill Cancer Patients with Opioid Refractory Pain

**DOI:** 10.7759/cureus.4811

**Published:** 2019-06-03

**Authors:** Eduardo E Lovo, Fidel J Campos, Victor E Caceros, Mario Minervini, Claudia B Cruz, Juan C Arias, William A Reyes

**Affiliations:** 1 Radiosurgery, International Cancer Center, Diagnostic Hospital, San Salvador, SLV; 2 Neurosurgery, International Cancer Center, San Salvador, SLV; 3 Pain Management, International Cancer Center, San Salvador, SLV; 4 Pallative Care, International Cancer Center, San Salvador, SLV

**Keywords:** radiosurgery, pain, hypophysys, oncology

## Abstract

Introduction

We report our initial series of terminally ill cancer patients treated with radiosurgery to the pituitary gland to alleviate pain.

Methods

A fully automated rotating gamma ray unit was used to deliver a high dose of radiation (150Gy) using an 8 mm collimator to the neurohypophysis in 11 patients suffering from opioid-refractory pain deriving from cancer.

Results

From November 2016 to November 2018, 11 patients were treated, and 10 were eligible for follow-up evaluation. Pain from bone metastases was present in 70%; others suffered from neuropathic and visceral pain. The median survival was 119.7 days (range: 32 to 370). The visual analogue scale (VAS) was nine (7-10) and standardized to 10; eight patients (80%) responded. The average VAS at the time of response was three (range: 1-6), and the average time to response was 2.8 days (range: 2-5). In the first week, 40% of the patients categorized the result as 'excellent', 30% deemed the result 'good', and 20% reported the result as 'poor'. One patient (10%) referred to the result as 'regular'. Those who responded were able to reduce their medications by at least 25%. The one-month average VAS score was five (range: 1-6), 60% reported a 'good' effect, 20% reported 'excellent' results, and 20% had no response. Of the study participants, 60% maintained their level of medicine consumption at lower than baseline. At the end of life, five patients (50%) presented substantial pain, two (20%) never had a therapeutic effect, and three (30%) died without substantial pain. There were no clinical complications that could be attributed directly to the treatment.

Conclusion

Radiosurgery to the pituitary gland is effective and safe and warrants further investigation to understand its potential role in palliative care in cancer patients.

## Introduction

Pain derived from cancer represents a heavy burden on patients, their families, and healthcare systems worldwide; even in economically advanced countries, refractory oncological pain is a challenge for palliative care [1-2]. Although there is no strict definition of 'refractory pain', some authors agree that chronic refractory pain in cancer is the persistent physical signs and symptoms associated with phenomena where surgical interventions, radiation, nerve blocks, physiotherapy, and opioids have failed (and are either not feasible or tolerated) [3]. These are terminally ill patients who are usually bedridden or whose mobility, daily activities, and quality of life are severely impaired directly by pain, sedation [1], or by the side effects caused by pain management treatments. Limited resource settings (as experienced in most of the world) impose a further toll on this set of patients among whom moderate to severe pain has been reported in more than half the population [4-5].

Hypophysectomy or induced lesions to the pituitary gland and the stalk have historically demonstrated adequate pain reduction in advanced cancer patients. Initially performed to prolong survival in hormonodependent metastatic cancer patients, more contemporary indications were focused mainly on alleviating pain [6-11]. The use of radiosurgery for this procedure dates to the studies by Hayashi and others who report attempts to alleviate pain in oncological and non-oncological patients. High levels (70% to 90%) of pain relief have been reported with apparently low complication rates [12-13]. There are current attempts to resurge this technique and to create randomized controlled trials to further validate its efficacy [14].

We report the first series of terminally ill patients that were treated for refractory oncological pain using stereotactic radiosurgery with a fully automated rotating gamma ray unit in a country categorized as a limited resource setting.

## Materials and methods

Patient selection

We conducted a prospective non-randomized trial of radiosurgical hypophysectomy for patients in palliative care suffering from cancer pain, refractory to opioid therapy in whom no reasonable alternative intervention therapy (such as surgery, radiation, or pain medication) was feasible. The trial was approved by the hospital’s ethical committee in November 2016. The inclusion criteria were patients with cancer-related pain categorized as severe (scoring 7-10) on the visual analog scale (VAS) despite the best medical algology practice and optimal opioid dosing, were 18 years or older with a life expectancy under six months, and their pain could derive from bone metastases or other organs affected by the disease. The cases were reviewed and approved by an ad-hoc committee that included a palliative care specialist and an algologist, oncologist, and radiation therapy and neurosurgery staff. The main endpoints of the study were the reduction of the VAS score by at least 50% and a reduction in opioid or pain medicine consumption.

Radiosurgical technique

**Figure 1:**
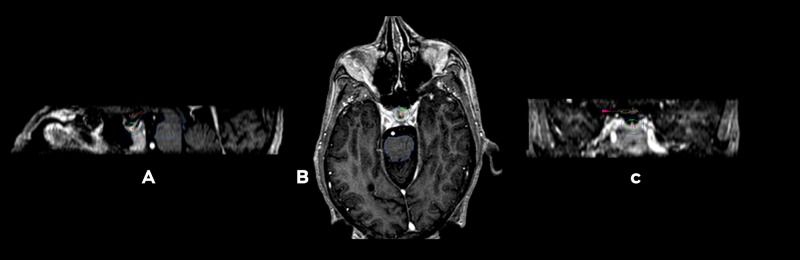
Main three-dimensional views Three-dimensional views of the dose distribution to the 8 mm collimator shot attempting to place the higher isodose lines to the most posterior part of the neurohypophysis. A. Sagittal view that shows the pituitary stalk and the gland being covered by the 50% isodose line in green corresponding to 75 Gy, exterior to it is the 25% isodose line in dark blue that corresponds to 37.5 Gy. B. Axial view. C. Coronal view, small pink arrow is signaling the visual pathway.

**Figure 2:**
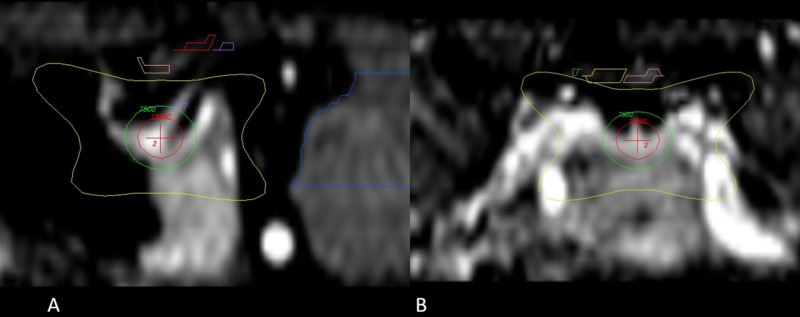
Main sagittal and coronal views of the region of interest and dose distribution A. Enlarged sagittal section of the hypophysis; the red cross signals the isocenter of the 8 mm shoot that is directed to the neurohypophysis, the red circle around the red cross is the 120 Gy isodose line. The green isodose line corresponds to the 75 Gy dose, and the most exterior line in yellow is the 10 Gy isodose line. B. Enlarged coronal section of the dose distribution above the yellow line or the 10 Gy isodose line; the optic pathway has been marked with yellow and pink.

**Figure 3:**
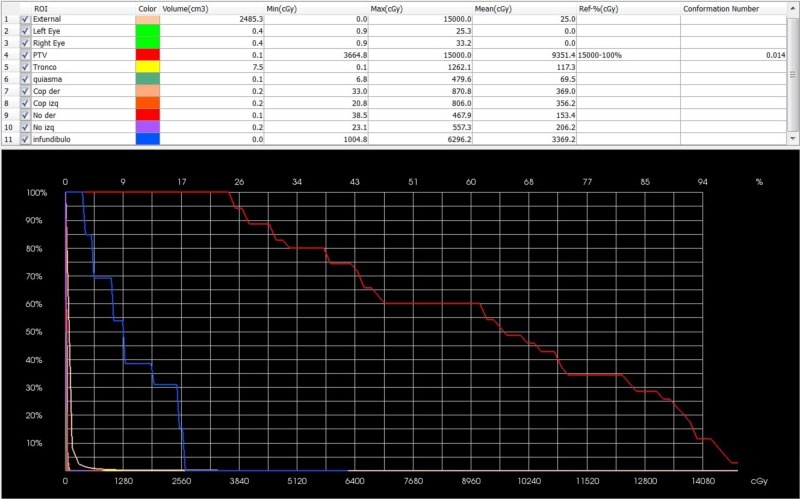
Screenshot of the dose volume histogram (DVH). From rows 6 to 10 is the optic pathway by components: chiasm, optic nerves, and optic tracts, Max(cGy) represents the maximum focal points not exceeding 8.7 Gy, Mean(cGy) is the average dose to the complete optic pathway 2.3 Gy. Brainstem, “Tronco” in yellow shows a focal point of a maximum dose of 12.6 Gy [Max(cGy)] and a mean dose of 1.1 Gy.

Patients fasted six hours on the day of the procedure and were prepared for sedation by the anesthesiologist using local anesthesia. An Infini stereotactic frame (Masep Medical Company, Shenzhen, China) was placed by a neurosurgeon, and the magnetic resonance imaging (MRI) acquisition was performed with a 1.5-tesla Avanto (Siemens Corporation. Erlangen, Germany). Normally, only one volumetric T1 of 1 mm to 1.5 mm slice thickness with no spacing of the head (apex to foramen magnum) was acquired; a contrast was added in one patient where metastatic lesions to the brain were also found. Images were transferred to the treatment planning station (TPS; Superplan; Masep Medical Company, Shenzhen, China). The organs at risk, which included the visual pathway and the brainstem, were contoured by neurosurgery; a single 8 mm collimator shot was placed to focus the isocenter of the shot on the neurohypophysis. Due to safety mechanisms specific to Superplan and the use of the prescribed high dose, we needed to place two shots in the same coordinates. The prescribed dose of 150 Gy to Dmax was administered to all patients with a gamma angle regularly fixed at 70°. The target was moved anteriorly or inferiorly as needed to comply with the organs at risk restrictions as stated by the American Association of Physicists in Medicine Task Group 101 Report [15] (Figures 1-3).

Patients did not receive more than 8 Gy as a threshold dose to more than 0.2 cc of the visual pathway. Patients did not receive a maximum point exceeding 10 Gy, nor did they receive over 10 Gy as a threshold dose to more than 0.5 cc of the brainstem or a maximum point exceeding 15 Gy.

Patient follow-up

Patients were contacted 72 hours after the procedure and every 15 days until the time of death. The VAS was standardized with patients and caregivers to 10 on the day of treatment to facilitate registry. Expected complications such as severe electrolytic variations, increased urine output, or visual disturbances were explained to caregivers as reasons to consult in case of an event. We used Wilcox signed rank test to determine p-values.

## Results

From November 2016 to November 2018, 11 patients were enrolled and treated with radiosurgical hypophysectomy for intractable pain (Table 1); four patients had prostate cancer, four had breast cancer, two had cervicouterine cancer, and one had lung cancer. Eight study participants (72%) had multiple lesions and pain mainly emerging from bone, two had visceral pain from peritoneal shedding, and one had intractable combined neuropathic pain from brachial plexus involvement and metastatic disease. None of the patients were receiving any form of active treatment for their cancer given their advanced disease staging made them ineligible for treatment. One patient was lost to follow-up. Thus, the results from the remaining 10 were registered. All patients were receiving opioids (80% morphine injections, 20% opiate derivatives) at the time of treatment with a mean of daily medicine intake for pain at 12.5 times per day (range: 10 to 15 times per day), including antidepressants, neuromodulators benzodiazepine, and non-steroidal regular pain medicine.

**Table 1:** Patient characteristics Abbreviation: VAS, visual analog scale.

Patient	Primary cancer	Bone metastases	Age (years)	VAS at treatment	VAS at response	Time elapsed for pain reduction (days)	Perception of treatment effect	Reduction of medicine and “pain rescue” at least 25%	VAS at one month	Perception of treatment effect at one month	Reduction of medicine and “pain rescue” at least 25% at one month	Died with pain	Pain recurrence before death	Time from treatment to death (days)
1	Prostate	Yes	78	10	2	2	Excellent	Yes	1	Excellent	Yes	No	0	38
2	Prostate	Yes	49	10	3	3	Good	Yes	5	Good	Yes	Yes	Last weeks	125
3	Breast	Yes	66	10	5	4	Regular	Yes	6	Good	Yes	No	0	34
4	Prostate	Yes	67	10	1	2	Good	Yes	3	Good	No	Yes	Last weeks	113
5	Cervix	No	58	10	10	0	Poor	No	10	Poor	No	Yes	Never went away	87
6	Lung	No	57	10	10	0	Poor	No	10	Poor	No	Yes	Never went away	93
7	Breast	No	44	10	2	2	Excellent	Yes	4	Good	No	Yes	Last days	128
8	Prostate	Yes	74	10	4	5	Good	Yes	5	Good	Yes	Yes	Last weeks	370
9	Breast	Yes	62	10	2	3	Excellent	Yes	1	Excellent	Yes	Yes	Last days	108
10	Prostate	Yes	92	10	3	3	Excellent	Yes	4	Good	Yes	No	0	30
Median		70%	64	10	3	3		80%	5		60%	70%		119.7

The average treatment time was 68.9 minutes (range: 63 to 89 minutes). One patient’s treatment lasted 159 minutes as multiple posterior fossa metastases where treated. The median survival was 119.7 days (range, 32 to 370 days). Mean VAS before treatment was nine (range: 7-10) and was standardized to 10 on the day of treatment. Eight patients (80%) presented a reduction of at least 50% of their initial pain, and the average VAS at the time of response was three (range: 1-6; p=0.013). The average time to response was 2.8 days (range: 2-5 days) after treatment, and 40% of the series categorized their result as 'excellent' (i.e., with minimal to no pain), 30% considered the treatment as 'good' (i.e., pain was adequately managed with medications), and 20% rated the treatment as 'poor' (no response). One patient (10%) referred to the treatment as 'regular' because the pain was alleviated less than 50% and there was significant pain despite the medications. Of those who responded, all reduced medications to five daily intakes (range, five to thirteen times per day; p=0.013) by at least 25% (actual reduction, 60%).

**Figure 4:**
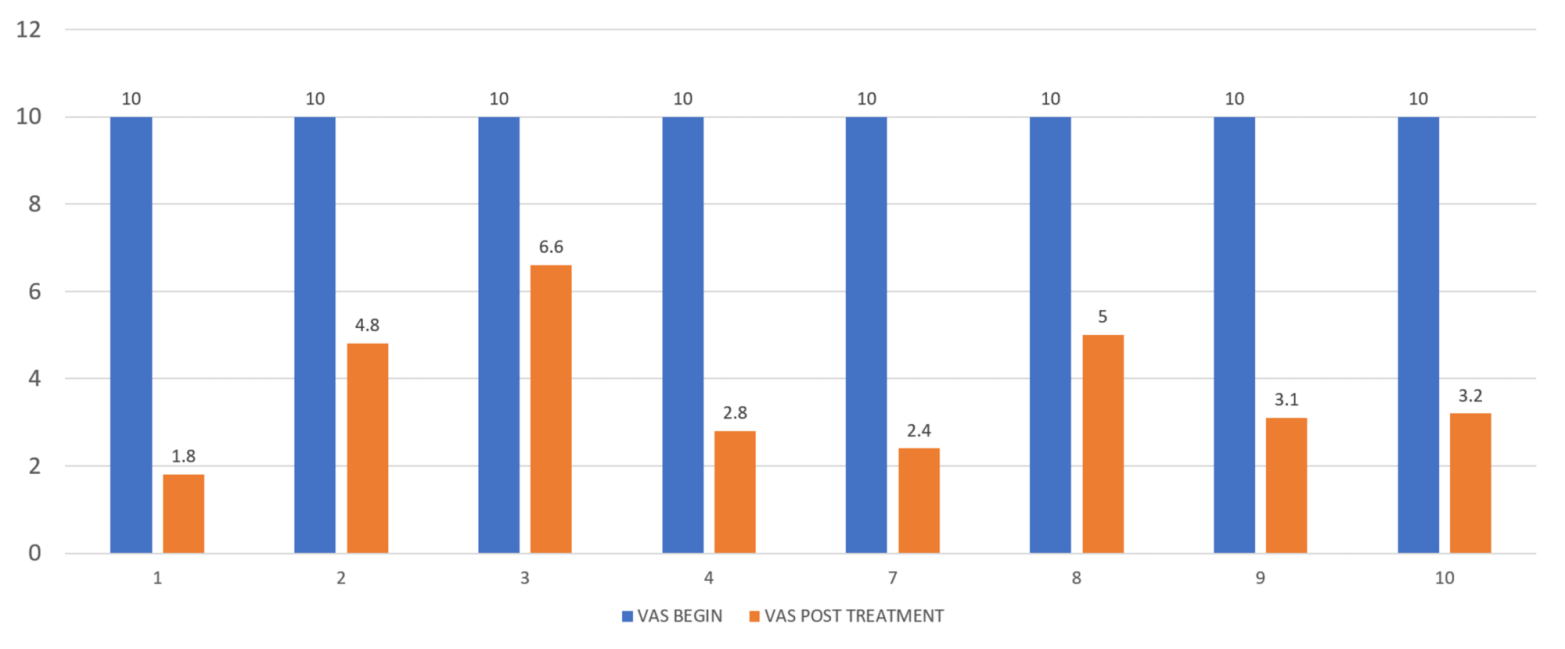
VAS before and at one month after treatment Mean perceived effect of radiosurgical hypophysectomy on pain at one month. Blue bar represents pain before treatment according to the standardized VAS and orange shows the VAS scores at one month for the eight patients who had a positive treatment effect. VAS: visual analog scale.

After 30 days, the VAS was five (range: 1-6; p=0.014), and 60% categorized the effect as 'good'. Among participants, 20% reported 'excellent' results, and 20% did not respond. At that time, 60% of the patients maintained their level of medicine consumption lower than baseline (mean: 7.3 times per day; range: 4-13 times per day; p=0.014) from the time of treatment, with at least a 25% reduction and an actual reduction from baseline at 41% for one month (Figure 4).

**Figure 5:**
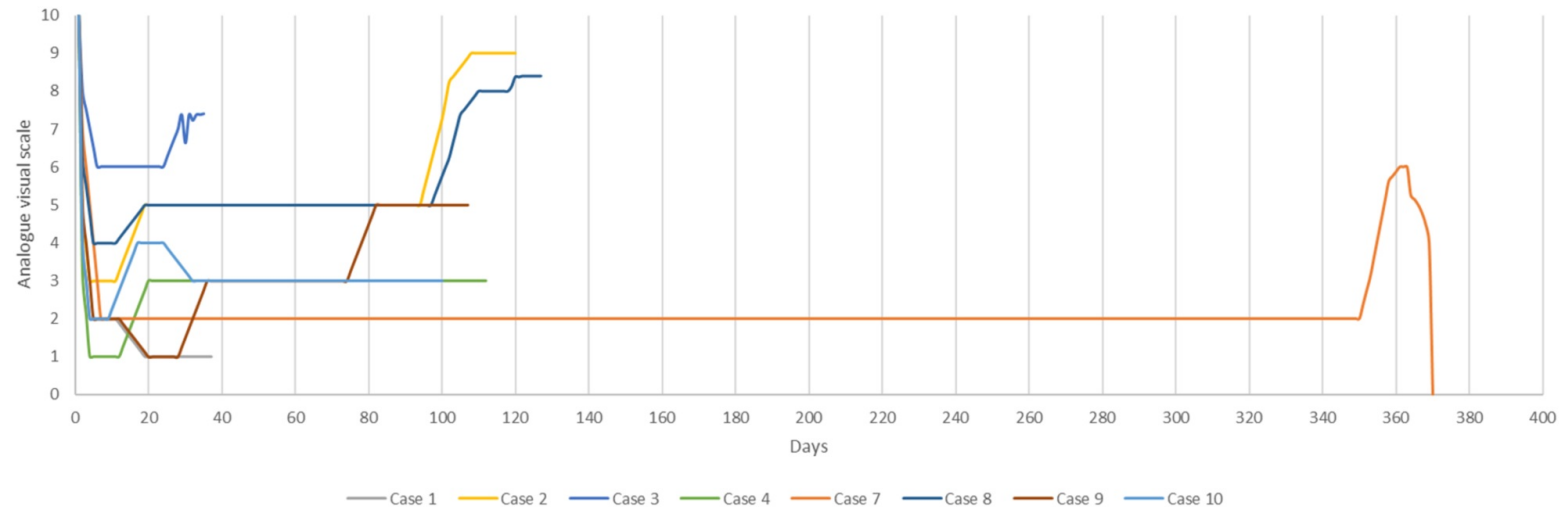
Perceived effect of radiosurgical hypophysectomy on pain, from the time of treatment until death The response of eight patients who had a positive treatment effect on the original scale of pain appear from the day of treatment until time of death. As shown, substantial pain recurred in most patients days or weeks before their death, with one patient achieved a long-lasting effect that reached almost one year.

At the end of life, five patients (50%) presented substantial pain (VAS 7-10), three of these patients presented it during the last days before death, two during the last weeks, two (20%) never had a therapeutic effect, and three (30%) patients died without substantial pain according to caregivers (Figure 5). 

## Discussion

Lesions to the pituitary gland to alleviate intractable pain have a long neurosurgical tradition, and the surgical technique has evolved to reduce morbidity [7-12]. Radiosurgery has developed into a noninvasive, safe, and effective technique that can attain results similar to more traditional surgical techniques. Although the underlying biological mechanism of the procedure remains unknown, Ramirez [11] argues that hormonal effects at the hypothalamus most likely explain the effect of pain relief. There is evidence that other surgical procedures such as oophorectomy and orchiectomy also provide pain relief, adding further support to the hormonal effect in pain perception or the elimination of hormonal feedback and its effect on pain-suppression at the hypothalamus [11]. Ramirez and others studied the effect of surgical hypophysectomy and the effect on the hypothalamic region, but such findings do not seem to explain how radiosurgery works. The quick-acting effect on pain relief after radiosurgical hypophysectomy goes beyond the eventual tumor response to hormonal alterations and pose even more complex questions as to the likely radiobiological effect of radiosurgery; it also possibly supports the idea of 'radiomodulation,' as the clinical effects are much faster than what is expected of tissue response or ablation to a high dose of radiation [16]. Our center directs the highest isodose towards the neurohypophysis. given that it stores oxytocin. High doses of radiation may redirect the oxytocin towards the dorsal horns of the spinal cord and supraspinal levels which have clear implications on pain modulation of neuropathic and inflammatory origin [17-19], and that could also explain the transitory pain relief effects on non-oncological pain such as thalamic pain and orofacial pain [20].

Refractory pain in cancer patients during their terminal phase is surely underrated worldwide but especially so in geographical settings with limited resources [4-5]. At best, where more efficient health and palliative care is available, patients are mainly managed by opiates that produce sedative states [1] or incur other well-known side effects [21] without necessarily producing an optimal pain control in some patients. Revitalizing well-known noninvasive techniques such as radiosurgical hypophysectomy could align with the palliative armamentarium to improve end-stage quality of life in selected patients. In the present series, a significant portion of the patients who survived past one month died with different degrees of pain and needed obvious support from regular pain management schemes as further investigation is warranted to identify factors associated with improved durability of pain. The patient that survived the longest (over one year) had prolonged pain control and only experienced pain in the weeks before his death. This patient and one other experienced opioid deprivation symptoms as they suspended their chronic use after feeling pain-free in the first week after treatment.

While there is a historical association between the best effects and patients with bone metastases, our series included patients without bone metastases because data for non-oncological pain and the initial efficacy described [20] could be potentially useful in patients with pain derived from cancer yet not emerging from diffuse bone involvement. The numbers in the present series are too small to dismiss this technique in such patients, but we must caution that two of the three patients without bone involvement were the two patients who never responded.

The present study was merely descriptive in a clinical sense. None of the patients had follow-up MRIs pretreatment or posttreatment hormonal measurements. Apart from potential clinically derived complications expected from high-dose radiation to the pituitary stalk (e.g., diabetes insipidus or frank visual alterations), we could not use hormonal screening to detect more subtle alterations that may result in potential adverse effects. Given this study limitation, further investigations warrant adequate laboratory studies attributed to opioid function and, eventually, posttreatment imaging.

Although rotating gamma ray units as a technique are lesser known than their original counterpart, the Gamma Knife, dosimetry studies have proven them to be equivalent [21]. These studies provided confidence for the safe delivery of a high dose of radiation close to sensitive, at-risk organs (e.g., the visual pathway and the brainstem) making radiosurgical hypophysectomy a clinical alternative for pain management in patients facing refractory oncological pain.

## Conclusions

This is the first series to describe the clinical results of radiosurgical hypophysectomy using a fully automated rotating gamma ray unit. The results as described by previous authors seem useful as an ancillary technique in the palliative care setting where every other alternative to alleviate pain is not viable. This procedure seems safe, and its effect over pain seems prolonged enough until weeks or days before death. Therefore, pain specialists should work closely with palliative care specialists to provide optimal care for these patients.
